# Specialisation and breast cancer survival in the screening era

**DOI:** 10.1038/sj.bjc.6600949

**Published:** 2003-05-27

**Authors:** D Kingsmore, A Ssemwogerere, D Hole, C Gillis

**Affiliations:** 1 University Department of Surgery, Western Infirmary, Dumbarton Road, Glasgow G11 6NT, UK; 2 Department of Public Health, University of Glasgow, 1 Lilybank Gardens, Glasgow G12 8RZ, UK

**Keywords:** breast cancer, specialisation, screening, survival

## Abstract

It is recommended that specialist surgeons treat all breast cancer, although the limited evidence to support this is based on treatment patterns prior to the introduction of screening. Whether a specialist survival advantage exists in the post-screening era is uncertain, as referral and treatment patterns may have changed, in addition to the effect of screening on the natural history of breast cancer. Our aim was to determine the impact of screening on the caseload and case-mix of specialist surgeons, to determine if the survival advantage associated with specialist care is maintained with longer follow-up and persists after the introduction of screening. Using the West of Scotland Cancer Registry, all 7197 women treated for breast cancer in a 15-year time period (1980–1994) in a geographically defined cohort were followed up for an average of 9 years, and pathological stage and socioeconomic status were linked with mortality data. We show that the caseload of specialists has increased substantially (from 11 to 59% of the total workload) and that smaller cancers have been selectively referred. However, even after allowing for pathological stage, socioeconomic status and method of detection, specialist treatment was associated with a significantly lower risk of dying (prescreening: relative risk of dying=0.83, 95% CI=0.75–0.92; post-screening: relative risk of dying=0.89, 95% CI=0.78–1.00). We conclude that this survival benefit is most consistent with effective surgical management rather than selective referral, the influx of screen-detected cancers or adjuvant therapies.

Many studies have shown that treatment of breast cancer differs widely between types of hospital, geographical area and caseload of treating surgeon (LeeFeldstein *et al*, 1994; Sainsbury *et al*, 1995a, 1995b). In an attempt to minimise variability, the Kings Fund (Kings' Fund Forum Consensus Statement, 1986) and other professional associations subsequently (National Institutes of Health Consensus Conference, 1991) produced guidelines of treatment. All these guidelines recommend a policy of referral to a designated breast cancer team or specialist. However, in contrast to the number of guidelines published recommending specialist treatment, only three studies have reported a survival advantage associated with specialist care (Sainsbury *et al*, 1995a; Gillis and Hole, 1996). These were based on treatment that may be outdated, had limited follow-up and were conducted in the prescreening era. It is unclear whether the survival advantage demonstrated was a temporary delay to disease progression or a ‘curative’ advantage that persists with longer follow-up. In addition, none of these studies could ascertain the nature of the survival advantage associated with specialist treatment. Possible causes may include more unified multidisciplinary treatment, greater use of adjuvant therapies, selective referral and better case-mix. Indeed, the very implementation of protocols of treatment has been reported to reduce variability in treatment and improve outcome (Winstanley *et al*, 1995).

Population-based screening was introduced in 1988. A series of quality assurance guidelines were produced to ensure appropriate treatment of small screen-detected cancers, and included recommendations on pathological analysis, referral to specialist centres, multidisciplinary meetings and protocols. Thus, both referral of these small screen-detected cancers and treatment patterns may have changed, in addition to any impact of screening on the natural history of breast cancer itself.

The aim of this study is three-fold: firstly, to establish the impact of screening on the caseload and case-mix of specialist surgeons. Secondly, to determine if the survival advantage demonstrated in the prescreening era is maintained with longer follow-up, and thirdly, to ascertain if the survival advantage associated with specialist treatment persists after the introduction of screening.

## PATIENTS AND METHODS

All women aged under 75 years with a histologically proven invasive breast cancer diagnosed between 1 January 1980 and 31 December 1994 were identified through the West of Scotland Cancer Registry. The study population was defined geographically using postcode sectors and formed the catchment areas for 10 hospitals and two screening centres (population base 1.5 million). Socioeconomic status was based on postcode sectors using the Carstairs' deprivation index (Carstairs and Morris, 1988).

In total, 7673 women were identified and the pathology reports of 7197 (94%) were reviewed. The following pathological details were recorded: tumour size, axillary lymph node status (number examined and number positive) and grade. Dates and causes of death were obtained from the Registrar General for Scotland and included all deaths up to 31 December 1997. The National Breast Screening Program started in June 1988 in the study area. Women with screen-detected cancers were identified from linkage with the breast screening centres that serve the population base.

Surgeons were selected as specialists for the present purpose, as in the previous study based on local surgeons' perception of who had a special interest in breast cancer, (Gillis and Hole, 1996). Our aim in this study was not to define a specialist, nor to determine the nature of a previously demonstrated survival advantage, but to ascertain if the concept of specialisation was applicable in the postscreening era. Specialist surgeons had several characteristics in common: the setting up of a dedicated breast clinic, a defined association with pathologists and oncologists, entry into clinical trials, and keeping a dedicated record of women with breast cancer under their care. These characteristics were not used as selection criteria, but clearly reflect a dedicated interest in breast cancer treatment.

Cox's proportional hazards model (Cox, 1972) was used to analyse survival patterns, allowing a relative benefit to be calculated after adjusting for differences in case-mix. Adjustment was made for age group (<50, 50–64, 65–74 years), socioeconomic status (Carstairs categories: 1,2=affluent; 3–5=intermediate; 6,7=deprived), pathological tumour size (<10, 10–19, 20–39, 40+ mm), nodal status (positive, negative), timeperiod (Period I=1/1/80–31/12/83, Period II=1/1/84–30/6/88, Period III=1/7/88–30/9/91, Period IV=1/10/91–31/12/94) and method of detection (symptomatic or screen-detected). The prescreening era consisted of time periods I and II (1/1/80–30/6/88), and the postscreening era of time periods III and IV (1/7/88–21/12/94).

The caseload of specialist surgeons was examined firstly as a percentage of the total workload each year. The caseload was then examined for the pre and postscreening eras, firstly by the contribution made by each age category to the total workload, and secondly by the percentage of women in each age category treated by specialists. The case-mix of the specialists and nonspecialists was then compared in each screening era by tumour size, nodal status and deprivation category. The 5- and 10-year survival rates for women treated by specialists and nonspecialist surgeons were estimated using the Kaplan–Meier technique. The hazard ratio for women treated by specialists relative to nonspecialists was compared for both the prescreening and post screening eras with adjustment made for tumour size, nodal status, age, deprivation category and method of detection.

## RESULTS

**Figure 1:**
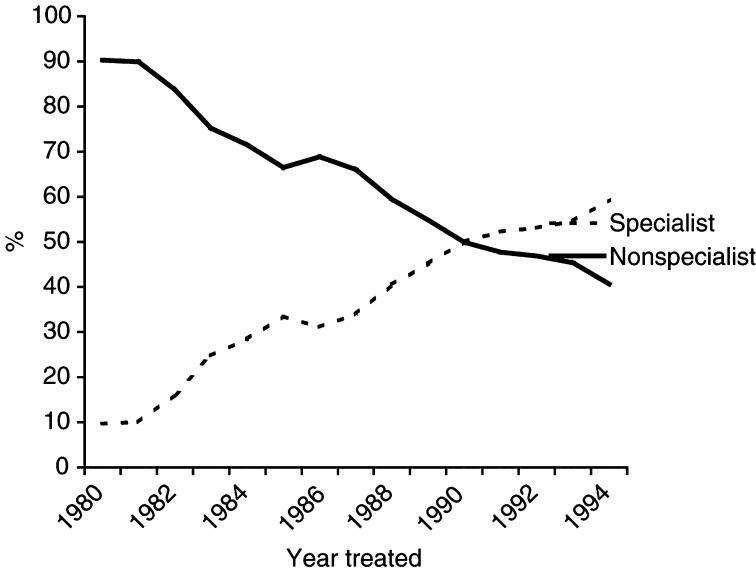
Percentage of all women treated by speciality of treating surgeon by year of diagnosis.

The percentage of women treated by specialists increased steadily from 11 to 59% of the total workload over the period of the study (Figure 1

). In the prescreening era, specialists treated more women in the intermediate age category (47 *vs* 41%), and nonspecialists saw more women in the older age category (24 *vs* 31%) (Table 1

**Table 1:** Caseload of specialist and non-specialist surgeons by time period and age

	Age	**Specialists**		**Nonspecialists**		**% treated by**
**Time period**	**(years)**	***n***	**%**	***n***	**%**	**specialists**
Prescreening						
	<50	269	29	796	28	25
	50–64	427	47	1184	41	26
	65+	221	24	889	31	20
						
Total		917	100	2869	100	24
						
Postscreening						
	<50	430	24	421	26	50
	50–64	980	55	717	44	58
	65+	369	21	494	30	43
Total		1779	100	1632	100	52

). Postscreening, the difference in the age distribution was more pronounced (women <50 years=55% *vs* 44%, 65+years=21% *vs* 30%). The percentage of all women in each age category treated by specialists doubled (<50 years from 25 to 50%, 50–64 years from 26 to 58%, 65+ years from 20 to 43%).

The case-mix and caseload were similarly analysed by tumour size, nodal status and deprivation category (Table 2

**Table 2:** Case-mix of specialist and nonspecialist surgeons by time period and age

		**Specialists**		**Nonspecialists**		
**Time period**	**Factor**	***n***	**%**	***n***	**%**	**% treated by specialists**
*Prescreening*						
Tumour size (mm)	<10	34	5	94	5	26
	10–19	180	25	534	26	25
	20–39	337	46	972	47	26
	40+	173	24	448	22	28
						*χ*^2^=0.75, *P*=0.39
Nodal status	Negative	387	50	837	46	32
	Positive	388	50	983	54	28
						χ^2^=3.24, *P*=0.07
Deprivation category	Affluent	231	25	554	19	29
	Intermediate	345	38	1366	48	20
	Deprived	341	37	949	33	25
						*χ*^2^=0.41, *P*=0.52
						
*Postscreening*						
Tumour size (mm)	<10	250	16	63	5	80
	10–19	549	36	441	33	55
	20–39	559	37	638	47	47
	40+	168	11	199	15	46
						*χ*^2^=90.90, *P*<0.001
Nodal status	Negative	848	59	639	55	57
	Positive	586	41	525	45	53
						*χ*^2^=4.54, *P*=0.03
Deprivation category	Affluent	446	25	294	18	60
	Intermediate	767	43	840	52	48
	Deprived	566	32	498	30	53
						*χ*^2^=5.42, *P*=0.02

). Prior to screening, the only significant difference in case-mix between specialists and nonspecialists was the distribution by socioeconomic status (specialists treating more women in the affluent and deprived categories), although the trend across the deprivation categories was not significant. However, in the postscreening era, specialists treated women with smaller cancers (<10 mm: 16 *vs* 5%, 10–19 mm: 36 *vs* 33%, 20–39 mm: 37 *vs* 47%, 40+mm: 11 *vs* 15%; *P*<0.001). The distribution of nodal status was also significantly different, although both groups treated a higher percentage of node-negative women postscreening.

The 5-year survival of all women, irrespective of who treated them, improved from 60% in the prescreening era to 73% postscreening (relative hazard ratio (RHR))=0.58, 95% CI=0.53–0.62, *P*<0.001 adjusted for age). This could not be accounted for by changes in patterns of treatment, tumour size, nodal status, deprivation category, screening status or age-distribution (RHR=0.77, 95% CI=0.71–0.83, *P*<0.001, after allowing for these variables).

The survival advantage associated with specialist treatment of women in the prescreening era persists with the addition of 4 extra years of follow-up, compared with our previous publication (RHR=0.83, *P*<0.001, Table 3

**Table 3:** Survival of women treated by specialist and nonspecialist surgeons by time period

	**Specialists**			**Nonspecialists**		
**Time period**	**5 years**	**10 years**	**RHR**	**5 years**	**10 years**	**RHR**
*All cause mortality*						
Prescreening	67%	49%	0.83	58%	42%	1
			(0.75–0.92)			(Baseline)
Postscreening	77%		0.89	70%		1
			(0.78–1.00)			(Baseline)
*Breastcancer specific mortality* Prescreening	71%	64%	0.82	64%	52%	1
			(0.73–0.92)			(Baseline)
Postscreening	81%		0.89	75%		1
			(0.77–1.02)			(Baseline)

RHR=Relative hazard ratio (95% confidence limits), adjusted for tumour size, age group, socioeconomic status, year of diagnosis and method of detection.

). This benefit was also seen in the postscreening era (RHR=0.89, 95% CI=0.78–1.00, adjusted for tumour size, nodal status, deprivation category, screening status and age). However, the survival benefit associated with specialist treatment decreased in the postscreening era from a 17% reduction in the risk of dying (RHR=0.83 prescreening) to an 11% reduction (RHR=0.89 postscreening). Analysing breast-cancer-specific mortality did not alter this survival advantage.

The reduction in the risk of death associated with specialist treatment was seen in those women with tumours smaller than 4 cm (Table 4

**Table 4:** Survival of women treated by specialist and nonspecialist surgeons by tumour size, nodal status, age, socioeconomic status

	**5-year survival (%)**			
**Factor**	**Specialists**	**Nonspecialists**	RHR^*^	**95% CI**
*Tumour size* (cm)				
<2	89.9	81.6	0.78	(0.64–0.91)
2–3.9	76.6	68.5	0.79	(0.69–0.92)
4+	49.6	51.1	0.99	(0.83–1.19)
				Test for interaction, *P*=0.004
*Nodal status*				
Negative	88.7	84.1	0.82	(0.70–0.95)
1–3 nodes positive	73.9	63.3	0.75	(0.65–0.88)
4+ nodes positive	57.3	40.0	0.81	(0.69–0.88)
				Test for interaction, *P*=0.38
*Age group (years)*				
<50	75.7	69.5	0.79	(0.68–0.93)
50–64	78.4	65.7	0.83	(0.74–0.93)
64–74	73.6	66.8	0.89	(0.77–1.02)
				Test for interaction, *P*=0.38
				
*Socioeconomic status*				
Affluent	79.3	70.6	0.88	(0.74–1.04)
Intermediate	77.3	68.0	0.83	(0.74–0.94)
Deprived	73.8	63.5	0.82	(0.72–0.93)
				Test for interaction, *P*=0.70

Adjusted for tumour size, nodal status, age, method of detection and socioeconomic status; 95% CI=95% confidence interval.

). The survival benefit was maximal in women with tumours smaller than 4 cm (8% difference in 5-year survival), with 4+ nodes positive (17% difference in 5-year survival), in women aged 50–64 years (13% difference in 5-year survival), and in the most deprived women (10% difference in 5-year survival).

## DISCUSSION

This study has shown that while the caseload of specialists has steadily increased over a 15-year period and the case-mix has altered with the implementation of the national screening programme, the survival advantage associated with specialist treatment has persisted with longer-term follow-up (median 13 years) and is also apparent in the postscreening cohort of women (median follow-up 6 years).

In this and the previous study (Gillis and Hole, 1996), surgeons were chosen as specialists on the basis of local surgeons' opinions of who exhibited a particular interest in breast cancer. The specialists shared several common characteristics, for example, keeping separate records of women they treated for breast cancer, entry into clinical trials, and a multidisciplinary approach to treatment. While these are vague characteristics, it must be remembered that at the time of that study there was no subspecialisation of surgical services. Indeed, the definition of ‘specialisation’ remains very variable. Other studies have used hospital type (McArthy and Bore, 1991; Basnett *et al*, 1992), caseload (Sainsbury *et al*, 1995a), affiliation with oncological services (Scorpiglione *et al*, 1995) and membership of local associations (Sainsbury *et al*, 1995b). While these criteria may allow simple differentiation into contrasting groups, they are also indirect measures of adequate and appropriate treatment. Thus, we feel that although our definition is subjective and not widely applicable, it has validity, being based on the opinion of peers, and accurately represents the focused training, dedicated interest, awareness of current treatment and concentration of service provision that underlies the premise of better treatment with specialisation.

The caseload of specialists doubled in the time period studied. Unexpectedly, this increased workload was not due solely to an influx of screen-detected cancers, but a steady incremental growth that started before screening. Although the pre-screening case-mix between specialists and nonspecialists was the same, after screening there was selective referral to specialists of younger women and smaller, screen-detected tumours. Almost half of all women treated by specialists in the age group 50–64 years, the age group offered screening, were screen-detected. However, it is interesting that in this same age group, 14% of women treated by nonspecialists had also been screen-detected. This re-enforces our premise that specialisation is not easily categorised, and that simply defining categories, for example, teaching hospitals *vs* district hospitals, does not necessarily correlate with quality of treatment.

Survival differences between the two groups of surgeons could be accounted for by differences in tumour size (lead-time bias) and also by method of tumour detection (length-time bias). Length-time bias was a possible confounding factor as screening may preferentially detect slower growing, more favourable tumours (Duffy *et al*, 1991; Klemi *et al*, 1992). This was confirmed, as the method of detection remained a highly significant variable in the survival analysis after allowing for all other factors (RHR=0.59, *P*<0.001).

We examined survival by two well-recognised methods to allow for the difference demonstrated in case-mix between specialists and nonspecialists. Firstly a multivariate analysis was performed and variables of tumour size, nodal status, age, method of detection and deprivation category were included as independent variables. Secondly, the data were stratified according to the above variables with the others included as independent variables. The benefit associated with specialist treatment was maximal in smaller, node-positive tumours, but also seen in all ages and socioeconomic groups. Given the size of the survival differences and the groups in which it is maximally seen, the survival benefit would not therefore be consistent with purely differential prescription of chemotherapy or endocrine therapy.

It is interesting to note that the survival benefit was not seen in tumours larger than 4 cm. Recent evidence suggests that local treatment and thus local recurrence may lead to dissemination of disease and poorer survival (Overgaard *et al*, 1997; Ragaz *et al*, 1997). It is very unlikely that tumours greater than 4 cm would have been suitable for or treated by breast conserving surgery. Thus, it is unsurprising that there is no difference in survival between specialists and nonspecialists in women with large tumours, as both will have been equally treated with mastectomy. Furthermore, women with advanced tumours are more likely to have disseminated disease at the time of diagnosis, and thus it is logical that any survival difference caused by local treatment will be overwhelmed by the overall poorer survival in this group of women.

Overall, 5-year survival between the two time periods has improved considerably (from 60 to 73%). The cause of this improvement is hard to determine, but could not be accounted for by changes in referral patterns, screening or case-mix in the multivariate analysis. It is unlikely that this can be solely because of increasing use of chemotherapy or endocrine therapy given the magnitude of this improvement.

We conclude that while the nature of this survival benefit remains elusive, we are confident that it is real, persistent, and most consistent with effective surgical management rather than selective referral, the influx of screen-detected cancers, or adjuvant therapies.
